# Real-World Results in Treating Diabetic Macular Edema With Faricimab at a London-Based Tertiary Eye Hospital

**DOI:** 10.7759/cureus.75002

**Published:** 2024-12-02

**Authors:** Moussa Al-Rufayie, Filomena Palmieri, Aseel Hamoud Bedan, Saad Younis, Ahmad Ali, Mathew Kurumthottical, Teerajet Taechameekietichai, Lorenzo Fabozzi

**Affiliations:** 1 Ophthalmology, Western Eye Hospital, Imperial College Healthcare NHS Trust, London, GBR

**Keywords:** angiopoietin-2, anti-vegf treatment, best-corrected visual acuity (bcva), central subfield thickness, cst, diabetic macular edema (dme), diabetic macular oedema, faricimab, vegf-a

## Abstract

Diabetic macular edema (DMO) poses a significant risk to vision, primarily caused by the leakage of retinal vessels. Traditional treatments involve anti-vascular endothelial growth factor (VEGF) agents and corticosteroids, though responses vary, necessitating frequent treatments. This retrospective study at a London-based tertiary eye hospital evaluates the efficacy of faricimab, a bispecific antibody inhibiting angiopoietin 2 (Ang-2) and VEGF-A, in treating DMO. Seventy-six eyes from 60 patients were treated with intravitreal faricimab injections over six months. Participants were divided into treatment-naïve and previously treated groups. The primary outcomes measured were best-corrected visual acuity (BCVA) and central subfield thickness (CST). Treatment-naïve patients showed significant improvement in BCVA from 0.86 ± 0.31 to 0.23 ± 0.26 logarithm of the minimum angle of resolution* *(Log MAR) and a reduction in CST from 385.71 ± 103.59 to 299.14 ± 76.00 μm. Previously treated patients also demonstrated improvements, with BCVA enhancing from 0.43 ± 0.38 to 0.31 ± 0.34 Log MAR and CST decreasing from 427.00 ± 129.40 to 318.88 ± 89.40 μm. Few adverse events were noted, affirming the safety profile of faricimab. The study concludes that faricimab significantly improves visual and anatomical outcomes in DMO patients, supporting its potential as a reliable treatment with extended dosing intervals.

## Introduction

Diabetic macular edema (DMO) is a major vision-threatening complication of diabetes, characterized by fluid accumulation in the macula from leaking retinal vessels, leading to significant visual impairment [1]. Chronic hyperglycemia causes oxidative stress and the accumulation of advanced glycation end products (AGEs), damaging retinal vessels and increasing vascular permeability [2]. Elevated pro-inflammatory cytokines and vascular endothelial growth factor (VEGF) further exacerbate leakage and fluid accumulation [3].

Current DMO treatments include anti-VEGF agents and corticosteroids, alongside managing diabetes and hypertension [4,5]. Anti-VEGF agents like ranibizumab and aflibercept reduce vascular permeability but can cause complications such as increased intraocular pressure (IOP) and endophthalmitis, while corticosteroids like dexamethasone and triamcinolone reduce inflammation but can lead to cataracts, increased IOP, and endophthalmitis [6-9]. Responses vary due to disease severity and genetics, necessitating frequent treatments and highlighting the need for more targeted treatments [10,11].

Faricimab is a bispecific antibody inhibiting both Ang-2 and VEGF-A, addressing key DMO pathways [12]. VEGF-A is well-known for its role in increasing vascular permeability and promoting neovascularization, while Ang-2 contributes to vascular instability, inflammation, and leakage [13]. Blocking Ang-2 stabilizes the blood-retinal barrier and reduces inflammation while inhibiting VEGF-A decreases permeability and angiogenesis [13]. Clinical trials show that faricimab improves visual acuity and anatomical outcomes with a favorable safety profile and extended dosing intervals, thus reducing the number of visits to the clinic [14].

## Materials and methods

Study design

This retrospective study was conducted at a London-based tertiary eye hospital and involved the analysis of data from patients diagnosed with DMO who received intravitreal faricimab injections over a six-month period.

Study population and sample size

The study included 76 eyes from 60 patients clinically diagnosed with DMO. Patients were stratified into two groups based on their treatment history. The treatment-naïve group consisted of patients who had not received prior treatment for DMO. The previously treated group included patients with suboptimal responses to prior anti-VEGF therapies and/or dexamethasone implants.

Study measures

All participants received intravitreal injections of faricimab, administered as a 6 mg dose drawn from a 0.05 mL solution of 120 mg/mL. The administration followed a treat-and-extend protocol, with treatment intervals adjusted based on individual patient responses, observed efficacy, and tolerability at each assessment point over the six-month study period.

Comprehensive ophthalmological evaluations were conducted at baseline, after each loading dose, and at the six-month mark. Baseline assessments included best-corrected visual acuity (BCVA), IOP, slit lamp examination, posterior pole examination, optical coherence tomography (OCT), and fundus autofluorescence (FAF). BCVA was measured using the early treatment diabetic retinopathy study (ETDRS) chart, and IOP was assessed using an iCare tonometer. Slit lamp and posterior pole examinations were performed with either a 66D or 90D indirect fundus-viewing lens. OCT imaging was conducted on the SPECTRALIS HRA+OCT platform (Heidelberg Engineering, Heidelberg, Germany), while FAF was assessed using either the Optos ultra-widefield system (Nikon Co. Ltd, Japan) or the SPECTRALIS HRA+OCT platform.

Medical and ocular histories were reviewed, and concomitant medications were documented to identify potential confounding factors. During each follow-up visit, BCVA tests, IOP measurements, slit lamp examinations, and posterior pole evaluations were repeated using the same methodology. OCT scans were performed to monitor changes in macular thickness and detect any anatomical alterations in the retina.

Outcomes

The primary outcomes of the study were changes in BCVA, macular thickness measured by OCT, and changes in biomarkers indicating disease progression or regression. Secondary outcomes included recording complications or adverse events associated with faricimab injections.

Statistical analysis

Descriptive statistics were used to summarize patient demographics and baseline characteristics. Paired t-tests were utilized to assess changes in BCVA and macular thickness from baseline to six months. Adverse events were categorized and reported as frequencies. Statistical significance was defined as a p-value <0.05.

Ethics statement

This study was conducted in accordance with the principles of the Declaration of Helsinki. Ethical approval was obtained from the hospital’s institutional review board prior to data collection. Patient confidentiality was maintained, and all data were anonymized before analysis.

## Results

The study cohort comprised 60 patients, accounting for a total of 76 eyes treated for DMO at a London-based tertiary eye hospital. The mean age of the participants was 62.28 years. Of these, 60% (n = 36) were male, and 40% (n = 24) were female. The diabetes classification included 11.67% (n = 7) with T1DM and 88.33% (n = 53) with T2DM. The distribution of treatment history revealed that 46.05% (n = 35) of the eyes were treatment-naïve, whereas 53.95% (n = 41) had previously received intravitreal anti-VEGF and/or dexamethasone treatments.

Treatment-naïve group

Best-Corrected Visual Acuity

**Figure 1:**
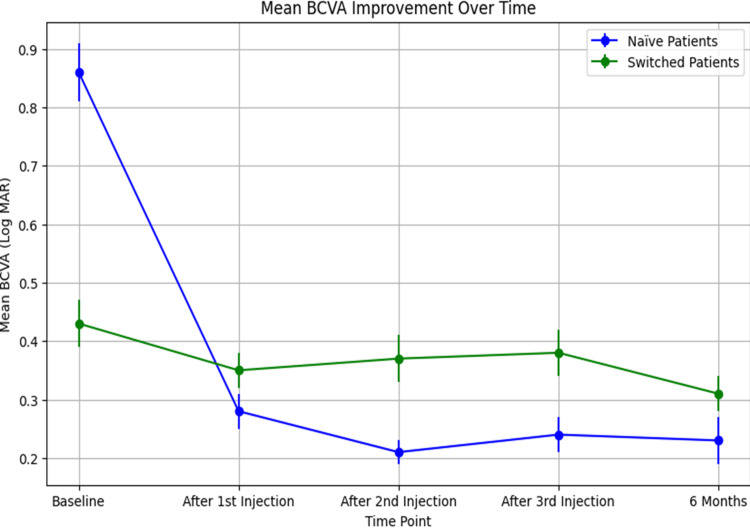
Mean BCVA improvement over six months for naïve (blue) and switched (green) patients treated with faricimab. BCVA, best-corrected visual acuity

There was a significant improvement in BCVA from baseline 0.86 ± 0.31 to 0.23 ± 0.26 logarithm of the minimum angle of resolution (Log MAR) at six months. Notable improvements were observed after each injection, with BCVA reaching 0.28 ± 0.31 Log MAR after the first injection and improving further to 0.21 ± 0.20 Log MAR after the second (Figure 1).

Central Subfield Thickness

**Figure 2:**
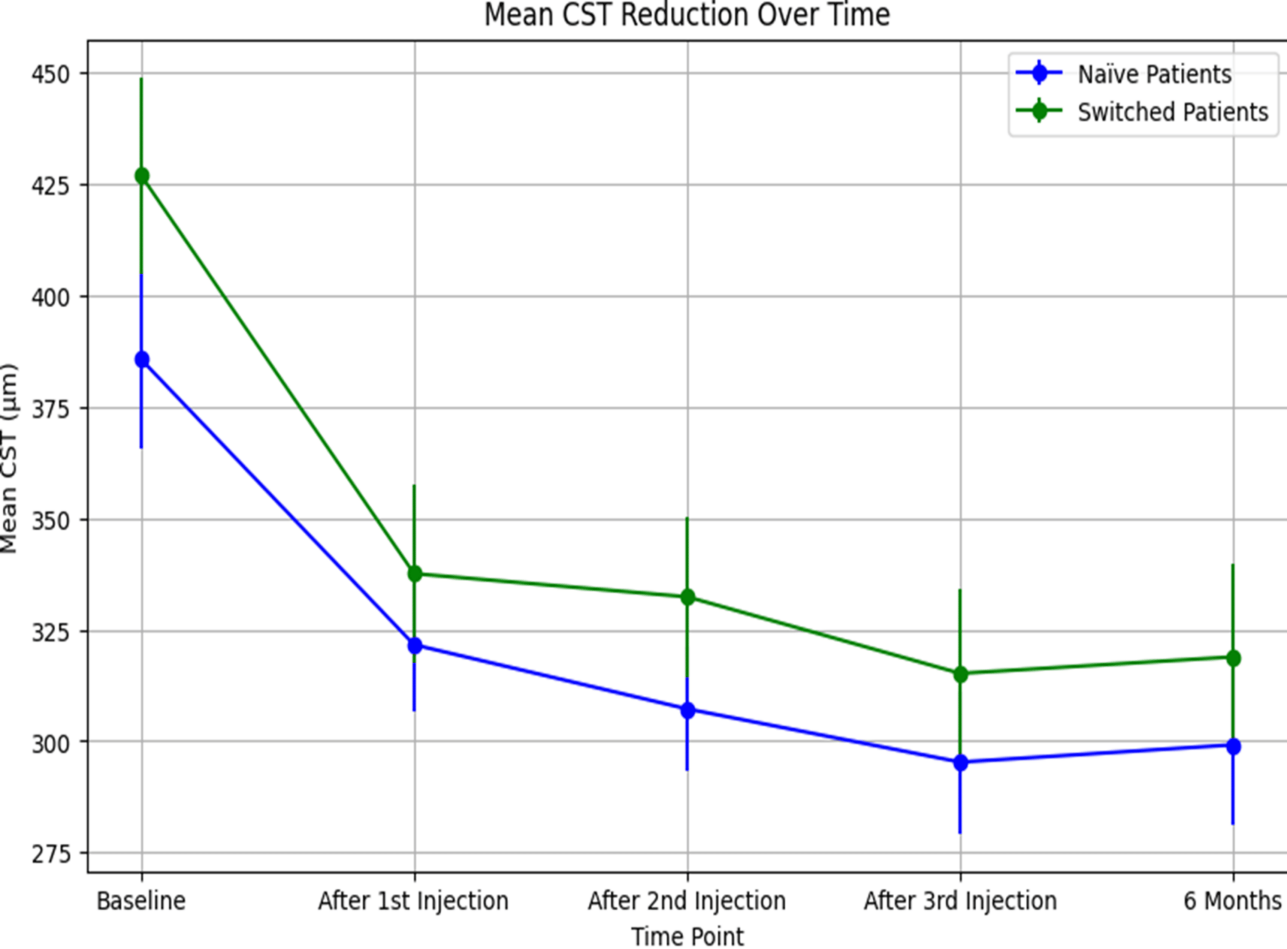
Mean CST reduction over six months for naïve (blue) and switched (green) patients treated with faricimab. CST, central subfield thickness

The initial mean CST was 385.71 ± 103.59 μm, reducing to 299.14 ± 76.00 μm by the end of the study period, indicating a reduction in macular thickness consistent with treatment efficacy (Figure 2).

Previously treated group

Best-Corrected Visual Acuity

Starting from a baseline of 0.43 ± 0.38 Log MAR, there was an improvement to 0.31 ± 0.34 Log MAR by the six-month follow-up. The BCVA slightly fluctuated after each injection but overall showed improvement (Figure 1).

Central Subfield Thickness

The initial average CST of 427.00 ± 129.40 μm decreased to 318.88 ± 89.40 μm after six months, with the most significant reduction occurring after the first injection (Figure 2).

Improvements were noted in both naïve and previously treated groups for biomarkers such as subretinal fluid, intraretinal fluid, macular hard exudates, ellipsoid zone disruption, and hyper-reflective foci. The epiretinal membrane was stable in patients who had it at baseline; it was observed in three naïve and six previously treated patients, with no new cases developing during the treatment period.

The study noted a few significant adverse events, including one treatment-naïve patient who experienced a retinal detachment after the third injection and another who suffered a mini-stroke following the second injection. Additionally, a patient from the previously treated group developed bilateral panuveitis after the fourth injection. These patients were subsequently excluded from the study.

## Discussion

The findings from this study demonstrate that faricimab significantly improves both BCVA and CMT in patients with DMO over a six-month period. The improvements observed in both treatment-naïve and previously treated patients highlight the efficacy of faricimab in diverse patient populations.

The results of our study align closely with those reported in other significant studies, such as the YOSEMITE and RHINE phase III clinical trials [15]. In our study, we observed substantial improvements in both BCVA and central macular thickness (CMT) over a six-month period in patients treated with faricimab. Specifically, BCVA improved from 0.86 to 0.23 Log MAR in treatment-naïve patients (p-value <0.05) and from 0.43 to 0.31 Log MAR in previously treated patients (p-value <0.05). Similarly, CST reduced from 385.71 to 299.14 μm in naïve patients (p-value <0.05) and from 427.00 to 318.88 μm in previously treated patients (p-value <0.05).

These findings are consistent with the results from the YOSEMITE and RHINE trials, which demonstrated that faricimab provided significant and sustained improvements in visual acuity and anatomical outcomes in patients with DMO. In these trials, faricimab was shown to maintain visual acuity gains with extended dosing intervals, reducing the treatment burden for patients compared to existing therapies.

Our study also noted a favorable safety profile for faricimab, with few significant adverse events. This is in line with the safety outcomes reported in the phase III trials, where faricimab was generally well tolerated, and the incidence of adverse effects was comparable to other anti-VEGF therapies. The rare complications observed, such as retinal detachment and mini-stroke, underscore the necessity for vigilant patient monitoring but do not diminish the overall positive benefit-risk ratio of faricimab treatment.

Our study has several limitations. As a retrospective study, it is inherently subject to selection bias and depends on existing medical records, which may be incomplete or inconsistent. The relatively small sample size and single-center design could limit how well the findings apply to wider populations. Furthermore, the six-month follow-up period does not provide insights into the long-term efficacy and safety of faricimab treatment. Future studies, including prospective designs with larger, multicentre cohorts and extended follow-up durations, are necessary to validate these results and assess long-term outcomes comprehensively.

## Conclusions

Overall, the consistency between our real-world findings and those from large-scale clinical trials highlights the efficacy and safety of faricimab in managing DMO. These results reinforce the potential of faricimab to become a key component in DMO therapy, offering significant clinical benefits with a manageable safety profile. Future studies with larger cohorts and longer follow-up periods will be essential to further validate these findings and explore the long-term impacts of faricimab treatment in diverse patient populations, with a particular focus on highlighting the reduced number of injections required over a longer time period.
